# Ursolic Acid Targets Glucosyltransferase and Inhibits Its Activity to Prevent *Streptococcus mutans* Biofilm Formation

**DOI:** 10.3389/fmicb.2021.743305

**Published:** 2021-09-27

**Authors:** Yucui Liu, Yanxin Huang, Cong Fan, Zhongmei Chi, Miao Bai, Luguo Sun, Li Yang, Chunlei Yu, Zhenbo Song, Xiaoguang Yang, Jingwen Yi, Shuyue Wang, Lei Liu, Guannan Wang, Lihua Zheng

**Affiliations:** ^1^National Engineering Laboratory for Druggable Gene and Protein Screening, Northeast Normal University, Changchun, China; ^2^State Key Laboratory of Microbial Technology, Shandong University, Qingdao, China; ^3^NMPA Key Laboratory for Quality Control of Cell and Gene Therapy Medicine Products, Northeast Normal University, Changchun, China; ^4^Guangdong Provincial Key Laboratory of Malignant Tumor Epigenetics and Gene Regulation, Department of Medical Research Center, Sun Yat-Sen Memorial Hospital, Guangzhou, China; ^5^Faculty of Chemistry, Northeast Normal University, Changchun, China

**Keywords:** ursolic acid, biofilms, extracellular polysaccharides, glucosyltransferases, mechanism

## Abstract

*Streptococcus mutans (S. mutans)*, the prime pathogen of dental caries, can secrete glucosyltransferases (GTFs) to synthesize extracellular polysaccharides (EPSs), which are the virulence determinants of cariogenic biofilms. Ursolic acid, a type of pentacyclic triterpene natural compound, has shown potential antibiofilm effects on *S. mutans*. To investigate the mechanisms of ursolic acid-mediated inhibition of *S. mutans* biofilm formation, we first demonstrated that ursolic acid could decrease the viability and structural integrity of biofilms, as evidenced by XTT, crystal violet, and live/dead staining assays. Then, we further revealed that ursolic acid could compete with the inherent substrate to occupy the catalytic center of GTFs to inhibit EPS formation, and this was confirmed by GTF activity assays, computer simulations, site-directed mutagenesis, and capillary electrophoresis (CE). In conclusion, ursolic acid can decrease bacterial viability and prevent *S. mutans* biofilm formation by binding and inhibiting the activity of GTFs.

## Introduction

Most oral diseases, including periodontal diseases and especially dental caries, are prevalent and common public health problems worldwide (Jakubovics et al., 2021). Approximately 60–90% of school-aged children and almost all adults suffer from dental caries (Wong et al., 2017). Dental caries is a chronic bacterial infectious disease that occurs on the tooth surface (Taubman and Nash, 2006). Traditional methods used to prevent or cure dental caries include mechanical removal of plaque (e.g., toothbrushing), the usage of broad-spectrum antibiotics, and more comprehensive forms of clinical treatment (e.g., root canal therapy, surgery, and dental restorations; Chen et al., 2016). However, the effect of prevention can be limited by poor toothbrushing technique and drug resistance (Cummins, 2013). Dental treatment is expensive, averaging 5% of total health expenditures and 20% of out-of-pocket health expenditures in most high-income countries, and is beyond the capacity of healthcare systems in most low- and middle-income countries (Vernazza et al., 2021). Therefore, more effective preventive and therapeutic strategies for dental caries are needed.

Dental caries is a typical biofilm induction-related disease, and cariogenic biofilms are one of the main factors leading to bacterial infection since they can protect microorganisms by enhancing microbial resistance to the host’s immunologic defense and antibacterial agents (Takahashi and Nyvad, 2011; Pitts et al., 2017). *Streptococcus mutans (S. mutans)*, the prime pathogen of dental caries, is an important contributor to the formation of cariogenic biofilms (Wang et al., 2020). *S. mutans* secretes three types of glucosyltransferases (GTFs), namely, GTF-I (also known as GtfB), GTF-SI (GtfC), and GTF-S (GtfD; Bowen and Koo, 2011). These GTFs can use dietary sucrose to synthesize extracellular polysaccharides (EPSs), and EPSs are the virulence determinants of cariogenic biofilms (Tamesada et al., 2004; Bowen and Koo, 2011; Jaña et al., 2018). GTF-I and GTF-SI catalyze mainly the synthesis of water-insoluble glucans, and GTF-S produces mainly water-soluble glucans (Monchois et al., 1999; Bowen et al., 2018). Water-insoluble glucans help bacteria adhering and aggregating on the tooth surface to form biofilms, while water-soluble glucan may supply a source of metabolizable carbohydrates for plaque bacteria and induce water-insoluble glucan formation (Jakubovics et al., 2021). Therefore, GTFs play key roles in causing and forming dental caries and are an effective target for the prevention and treatment of dental caries or other related diseases (e.g., infective endocarditis; Shun et al., 2005).

Ursolic acid, a type of pentacyclic triterpene compound, can be isolated in abundance from many foods (e.g., apples, olive, and basil) and medicinal plants (e.g., *Malus pumila*, *Ocimum basiliacum*, and *Rosmarinus officinalis*; Ikeda et al., 2008; Bacanlı et al., 2017; Hui et al., 2021). Ursolic acids have been reported to have many beneficial bioactivities, such as anticancer (Patlolla and Rao, 2012), anti-inflammatory (Pádua et al., 2014), antimicrobial (Kim et al., 2012), immunity regulation (Xu et al., 2019), and antiviral activities (Kong et al., 2013). In our previous research, we confirmed that the crude extract of *Bergenia crassifolia* leaves could inhibit *S. mutans* biofilm formation (Liu et al., 2017). In a screen of the active ingredients of *Bergenia crassifolia* leaves for antibiofilm activity, we found that ursolic acid had a significant effect. Here, we confirmed the antibiofilm activities of ursolic acid against *S. mutans* and further revealed the mechanism underlying the inhibitory effect of ursolic acid on GTF-mediated synthesis of EPSs. Our study provides a potential antimicrobial agent that can be used to prevent and cure oral and other GTF-related diseases.

## Materials and Methods

### Bacterial Strain, Growth Conditions, and Chemicals

*S. mutans* (ATCC 251175) was obtained from the Guangdong Microbiology Culture Center and was cultured in brain heart infusion (BHI) broth (Hopebio, Qingdao, China) supplemented with 1% sucrose at 37°C for 24h under aerobic conditions. After incubation, the bacterial concentration was 10^7^cfu/ml, as determined by spectrophotometry (OD_630_=0.2). Ursolic acid was purchased from the National Institutes for Food and Drug Control with purity >98% and was dissolved in dimethyl sulfoxide.

### Antimicrobial Activity Assay

The potential inhibitory activity of ursolic acid against *S. mutans* was determined by the microdilution method as described previously (Liu et al., 2017), with minor modifications. Briefly, ursolic acid was serially diluted twofold in BHI broth containing 1% sucrose, with the final concentration of ursolic acid ranging from 0.25 to 0.031mg/ml. Twenty microliters of sterile solution of resazurin sodium per well was added to the bacterial culture and incubated at 37°C for 24h. BHI broth alone was used as blank control, bacterial suspension alone was used as a noninhibition negative control, and chlorhexidine treatment at a final concentration of 0.6mg/ml was used as a positive control. As bacteria grow, resazurin is reduced to resorufin, resulting in the medium color changing from blue to pink (Palomino et al., 2002; Sarker et al., 2007). The lowest concentration of ursolic acid that could inhibit the medium color change from blue to pink was defined as the minimal inhibitory concentration (Palomino et al., 2002; Süntar et al., 2016). The bacterial cultures treated with ursolic acid at concentrations equal to or higher than the minimum inhibitory concentration (MIC) were transferred to BHI agar plates and incubated at 37°C for 24h. The lowest concentration that resulted in no visible bacterial colonies on the agar plates after incubation was defined as the minimal bactericidal concentration (MBC; Liu et al., 2017).

### XTT Reduction Assay

The effect of ursolic acid on the viability of biofilms was evaluated using a 2,3-bis (2-methyloxy-4-nitro-5-sulfophenyl)-2H-tetrazolium-5-carboxanilide (XTT) reduction assay as described previously (Duque et al., 2017), with some modification. Briefly, after 24h of incubation for cultured bacteria to form biofilms in 96-well microplates, supernatants were removed, and the wells were gently washed three times with PBS (pH 7.0) to remove nonadhered bacteria. Then, the cultures were incubated at 37°C for another 22h in 200μl of BHI broth including 100μl of ursolic acid at various concentrations. After removing the supernatants, 50μl of XTT (Sigma, St. Louis, MO, United States) reagent was added, and the microplate was kept in the dark for 2h at 37°C. Then, the absorbance of the colored product was detected at 490nm using an ELISA reader. Alternatively, after ursolic acid treatment, a crystal violet assay was used to evaluate the attachment of biofilm biomass as described previously (Cardoso Sá et al., 2012).

### Live/Dead Bacterial Staining

Fluorescence staining was used to determine biofilm integrity as described previously (Liu et al., 2017). After biofilm formation, ursolic acid was added at a final concentration of 1/2 MIC followed by incubation at 37°C for another 18h. The culture was then removed from the supernatant and gently washed twice with sterile water. Staining was carried out by means of the Live/Dead BacLight Bacterial Viability Kit (L13152, Invitrogen, Carlsbad, CA, United States) for 30min at room temperature in the dark. Stained cells were observed under a fluorescence microscope (Nikon Eclipse 80i; Nikon Co., Japan). The kit utilizes a mixture of SYTO 9, a green-fluorescent nucleic acid stains and propidium iodide (PI), a red-fluorescent nucleic acid stains. SYTO 9 is a membrane-permeable fluorescent marker that stains all cells, while PI penetrates only bacteria with damaged membranes and stains dead cells red. Twenty fields per sample were randomly selected to analyze the intensity of red and green fluorescence.

### Extracellular Polysaccharide Production Assay

The method for quantification of EPS production was conducted as described previously (Packiavathy et al., 2012). Briefly, the bacterial culture was incubated with or without ursolic acid at concentrations ranging from 0.008 to 0.125mg/ml at 37°C for 16h, the culture was centrifuged (4°C, 12000×*g*, 30min), and the supernatants and cells were collected. The water-soluble and water-insoluble glucans were prepared using the method described previously (Yano et al., 2012). Briefly, water-soluble glucans were obtained by ethanol precipitation of the supernatants. The cells were resuspended in 1M NaOH and centrifuged to collect supernatants, which were used to prepare water-insoluble glucans by ethanol precipitation. The phenol/H_2_SO_4_ method was used to quantify two types of EPS in the supernatant as described previously (Liu et al., 2017). The absorbance of the color was detected at 490nm using an ELISA reader.

### Molecular Dynamics Simulations

The crystal structure of glucansucrase was downloaded from the Protein Data Bank (ID: 3AIC) as the receptor, and the preparation work was done using the software Gold 5.2 and AutoDockTool 1.5.6. The MD simulation of the system was performed for 20ns under the npt ensemble, and the data were saved every other 5ps. CPPTRAJ was used for data analysis. The MMPBSA.py module was used to compute the binding free energy between the protein and ligand.

To further confirm the binding site between GTF-SI and ursolic acid, the amino acids in the predicted binding site were replaced by other amino acids, as shown in Supplementary Table S1 Afterward, the mutant GTF-SI proteins were subjected to molecular docking under the same conditions as above.

### GTF Activity Assay

The method for analyzing the enzymatic activity of the crude extract of GTFs was used as described previously by Koo *et al* (Koo et al., 2000). A 20-mL bacterial suspension of *S. mutans* was incubated in 200ml of BHI broth containing 1% sucrose at 37°C. After incubation, supernatants were collected, and ammonium sulfate was added at 60% saturation for 24h to prepare the protein. The crude enzymes were dissolved in PBS (pH 6.0). The reaction system mixture was the same as that described previously (Liu et al., 2017). The final concentration of ursolic acid ranging from 0.04 to 0.12mg/ml was used to measure the inhibition of the synthesis of EPSs. After incubation, the method to determine the amount of the two types of glucans was applied as described above.

### Preparation of Recombinant GTF and Its Mutants

cDNA fragments of wild-type GTF-SI (GenBank: M22054.1) and two mutant GTF-SI variants (Supplementary Table S1) were synthesized and inserted into the *Nde*I and *Xho*I sites of pCold I to generate pCold-GTF-SI recombinant plasmids with a 6× histidine tag at the N-terminus of the proteins (Ito et al., 2011). The recombinant proteins were purified by using GE metal affinity resin and dialyzed. SDS-PAGE was used to analyze recombinant GTF-SI proteins (shown in Supplementary Figure S1).

### Capillary Electrophoresis Assay

Capillary electrophoresis (CE) was used to quantify fructose production catalyzed by GTFs as described previously (Rizelio et al., 2012). Recombinant GTF-SI was crudely extracted from recombinant transformants by ultrasonic disruption. Then, 50μl of the crude enzyme solution together with or without ursolic acid at a final concentration of 0.03mg/ml or 0.02mg/ml was added to 1ml of sucrose (0.1M) and incubated at 37°C for 2h. After incubation, the culture was centrifuged (4°C, 12000×*g*, 10min), and the supernatants were collected and filtered with a 0.22-μm nylon membrane before being subjected to the CE assay. The CE assay was conducted in a CE system, and the conditions to detect fructose were as follows: The detection wavelength was set at 254nm; the temperature was maintained at 25°C; and fused silica capillaries had dimensions of 50cm in total length and 40cm in effective length, and the inner diameter and outer diameter were 50μm and 375μm, respectively. The sample injection was maintained at an elevation difference of 20cm from the nearest detector for 5s, and the separation voltage was 25kV. The capillary system was rinsed with 0.1M NaOH for 5min, distilled water for 3min, and PBS for 3min before each run.

CE can also be used to detect the binding of drugs and proteins, which was used in this study to analyze the interaction of ursolic acid with GTF-SI and its variants as described previously (Liang et al., 2020). GTF-SI and its variants were dissolved in PBS (20mm, pH=6.8) and filtered with a 0.22-μm nylon membrane. Ursolic acid was dissolved in the same solution as that used to dissolve the proteins, and the final concentration was 0.3mg/ml. Afterward, the GTF or its variants were incubated with ursolic acid for 2h. Finally, CE was conducted as described above with a few differences: The detection wavelength was 210nm, and the separation voltage was 20kV.

## Results

### Effects of Ursolic Acid on Biofilms

**Figure 1:**
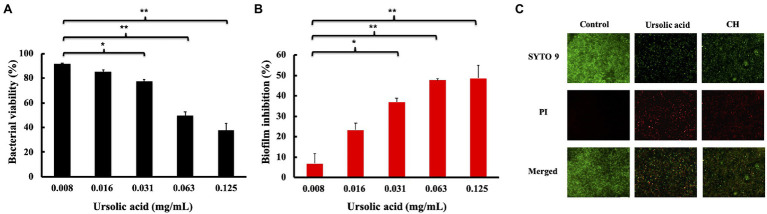
Effects of ursolic acid on biofilms. **(A)**: Percentage of bacterial viability within biofilms detected by XTT assay; **(B)**: Inhibitory percentage of ursolic acid on biofilm formation detected by crystal violet staining. Data are presented as the mean±standard deviation. ^*^p<0.05 and ^**^p<0.01. **(C)**: Effects of ursolic acid on biofilm structure. SYTO 9: green fluorescence, which stains both the dead and live bacterial cells; PI: red fluorescence, which stains dead bacterial cells. Control: ursolic acid untreated biofilms; CH: chlorhexidine (the positive control).

Biofilms are the key factors in the induction of dental caries and periodontitis. We first measured the MIC and the MBC values of ursolic acid against *S. mutans*. The MIC value is the lowest concentration that could prevent the culture color from changing blue to pink. The MBC value is the lowest concentration that resulted in no visible bacterial colonies on the agar plates after incubation. As shown in Supplementary Figure S2 and shown in Table 1, both the MIC and MBC values of ursolic acid against *S. mutans* were 0.25mg/ml. Based on the concentration of antimicrobial activities, we evaluated the effect of ursolic acid on *S. mutans* biofilms by the XTT reduction method. As shown in Figure 1A, the bacterial viability within the biofilms decreased as the concentration of ursolic acid increased, while the difference was most significant at the concentration of 0.063mg/ml. Compared with the positive control, ursolic acid showed similar inhibitory effects on the bacterial viability of biofilms at 0.125mg/ml (Supplementary Figure S3). In addition, the crystal violet staining results showed that the amount of biofilm was decreased upon ursolic acid treatment, as shown in Figure 1B, which is consistent with the data of the XTT reduction assay.

**Table 1:** The MIC and MBC values of ursolic acid against *S. mutans*.

Compound	Species/Strain	
	*S. mutans* (ATCC 25175)	
	MIC (mg/mL)	MBC (mg/mL)
Ursolic acid	**0.25**	**0.25**

*Negative control: the bacterial suspension alone*;

*Positive control: chlorhexidine at a final concentration of 0.6mg/ml*.

To observe the direct effects of ursolic acid on biofilms, the Live/Dead BacLight Bacterial Viability kit was used to examine *S. mutans* bacteria within the biofilm, as shown in Figure 1. Compared with the control sample, the sample treated with ursolic acid had a large number of red-stained bacterial cells, indicating dead cells. Furthermore, the biofilms were thin. This result confirmed that ursolic acid could significantly destroy biofilms by affecting bacterial survival and adhesion.

### Effects of Ursolic Acid on GTF Activity

**Figure 2:**
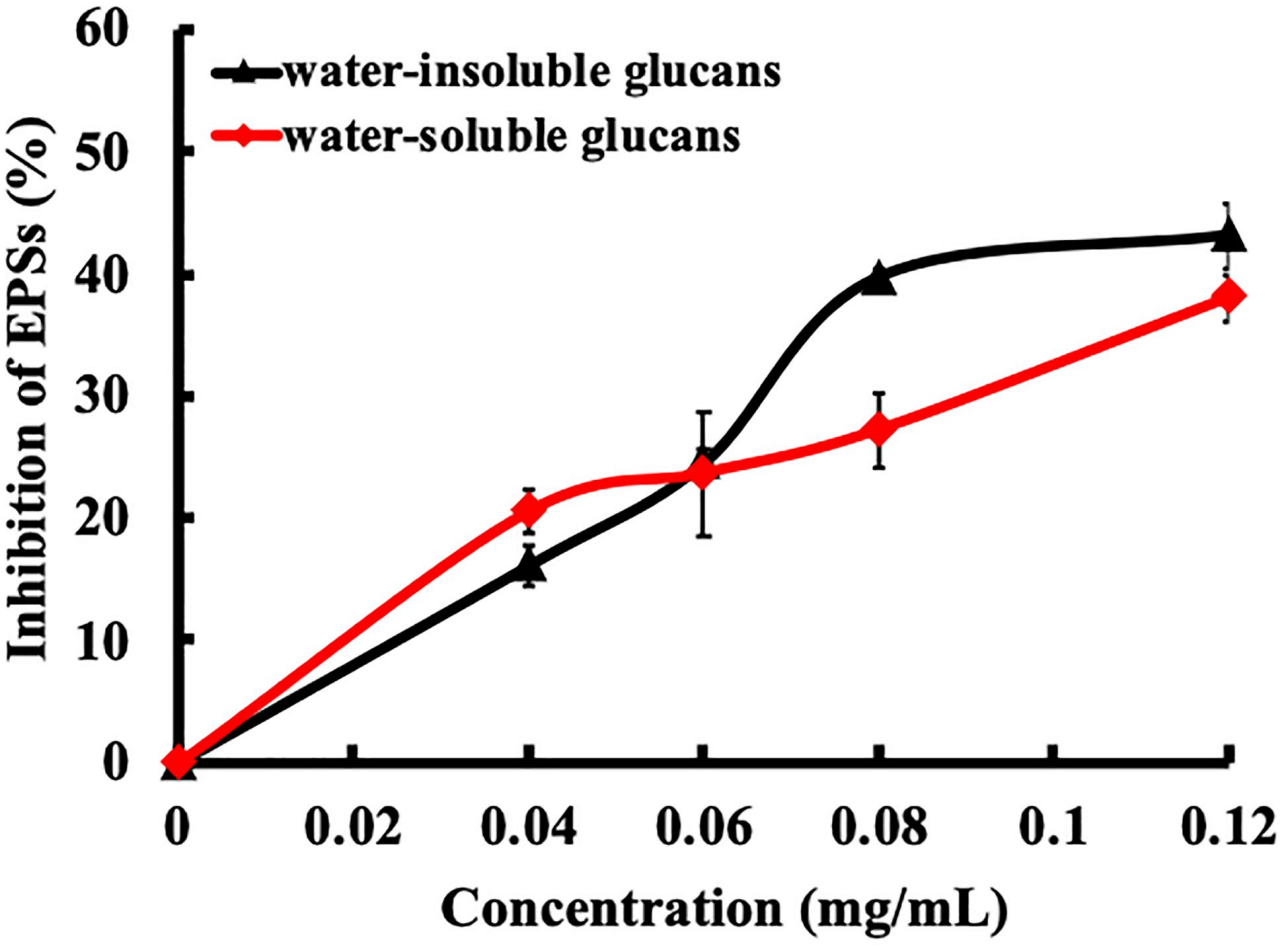
Effect of ursolic acid on glucosyltransferase (GTF) activities. GTFs were crudely extracted from bacterial culture medium and were then used to test the effect of ursolic acid on its activities by using an *in vitro* enzymatic reaction system. The final concentration of ursolic acid ranged from 0.04 to 0.12mg/ml. Extracellular polysaccharides (EPSs), including soluble and insoluble glucans, which are the catalytic products of GTFs, were measured as indicators of GTF activity. The data are presented as the mean ± standard deviation.

EPSs are the key factors contributing to bacterial colonization, biofilm formation, maturation, and caries. Because EPSs are synthesized by GTFs, we explored the effect of ursolic acid on GTF activity in crude bacterial extracts in the presence of sucrose *in vitro*. As shown in Figure 2, ursolic acid inhibited the activity of GTFs, resulting in a decrease in the synthesis of EPS products. The inhibition of ursolic acid on water-soluble glucan production was stronger than that on water-insoluble glucan production at low concentrations of less than 0.06mg/ml, while this inhibition tendency was reversed at concentrations from 0.06mg/ml to 0.12mg/ml. Combined with the data above, these results suggest that ursolic acid could suppress the aggregation or adhesion of *S. mutans* to form cariogenic biofilms by preventing GTFs from synthesizing EPSs.

### The Mode of Interaction Between Ursolic Acid and GTF-SI

**Figure 3:**
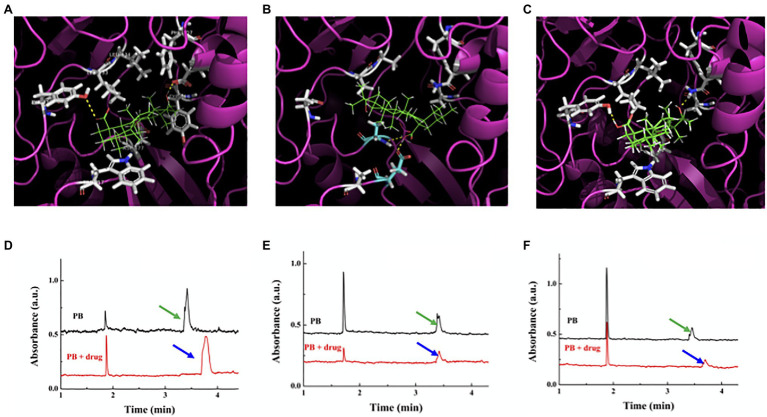
Interaction between ursolic acid and GTF-SI. **(A-C)** Binding model of ursolic acid with GTF-SI and its variants simulated by Gold software. (A) Wild-type GTF-SI; (B) GTF-SI variant A; (C) GTF-SI variant B. Ursolic acid, as the ligand, is shown in green. The protein is shown as a cartoon with pink color. The yellow dotted lines represent hydrogen bonds. **(D-F)**: Interaction between ursolic acid and GTF-SI measured by CE assay. The black line represents the migration time of GTF-SI and its variants, whereas the red line is the migration time of GTF-SI or its variants together with ursolic acid. (D) Wild-type GTF-SI; (E) GTF-SI variant A; (F) GTF-SI variant B. Arrows point to the peak of the protein (black)/protein and ursolic acid complex (red).

The catalytic center of GTF-SI has been shown to include two subsites for EPS synthesis: Subsite+1 contains the key amino acids for sucrose to bind, and subsite-1 contains the key amino acids for glucosyl moiety polymerization to form EPSs (Ito et al., 2011). In particular, Trp517 provides a platform for the acceptor glycosyl moiety of sucrose, and Tyr430 participates in hydrophobic interactions with carbon atoms of the glycosyl moiety in subsite+1; however, Asp909 and Tyr916 are related to recognition of the glucosyl moiety of the primary sucrose and formation of the glycosyl-enzyme intermediate in subsite-1 (Ito et al., 2011). As shown in Figure 3A, the simulation data showed that ursolic acid formed hydrogen bonds with Tyr430 and Asp909 of GTF-SI and had hydrophobic interactions with Leu433, Leu434, Phe907, Trp517, and Tyr916, which are the key sites for catalyzing sucrose to synthesize EPSs. Furthermore, the MD simulation results showed that the binding free energy of GTF-SI to ursolic acid was similar to that of sucrose (Supplementary Table S2). Then, we made two mutant GTF-SI variants in which the predicted amino acids that are essential for ursolic acid binding were replaced with other amino acids (Supplementary Table S2) to further verify the binding indicated by the simulation. The simulation showed that the number of amino acids in either GTF-SI variant that formed hydrogen bonds and hydrophobic interactions with ursolic acid decreased compared with that in wild-type GTF-SI (Figures 3B,C), and the interaction and binding free energy of the GTF-SI variants with ursolic acid were also decreased (Supplementary Table S2, S3).

Then, CE technology was used to further determine the interaction between GTFs and ursolic acid *in vitro*. As shown in Figure 3D, compared with that of the control, the migration time of the protein was prolonged by the addition of ursolic acid, which indicated that ursolic acid could bind with GTF-SI to change the charge-mass ratio, leading to a migration time-shift. The CE results further showed that the retention time of the variant A or variant B proteins was shorter than that of wild-type GTF-SI after the addition of ursolic acid, suggesting that the interaction between GTF-SI and ursolic acid became weaker or even lost as several key amino acids at the binding site were replaced (Figures 3E,F). These results indicate that ursolic acid might inhibit the enzymatic activity of GTFs through direct binding and that Phe907, Asp909 and Tyr916 in GTF-SI might be the key amino acids responsible for ursolic acid binding.

### Effects of Ursolic Acid on EPSs and Fructose Production

**Figure 4:**
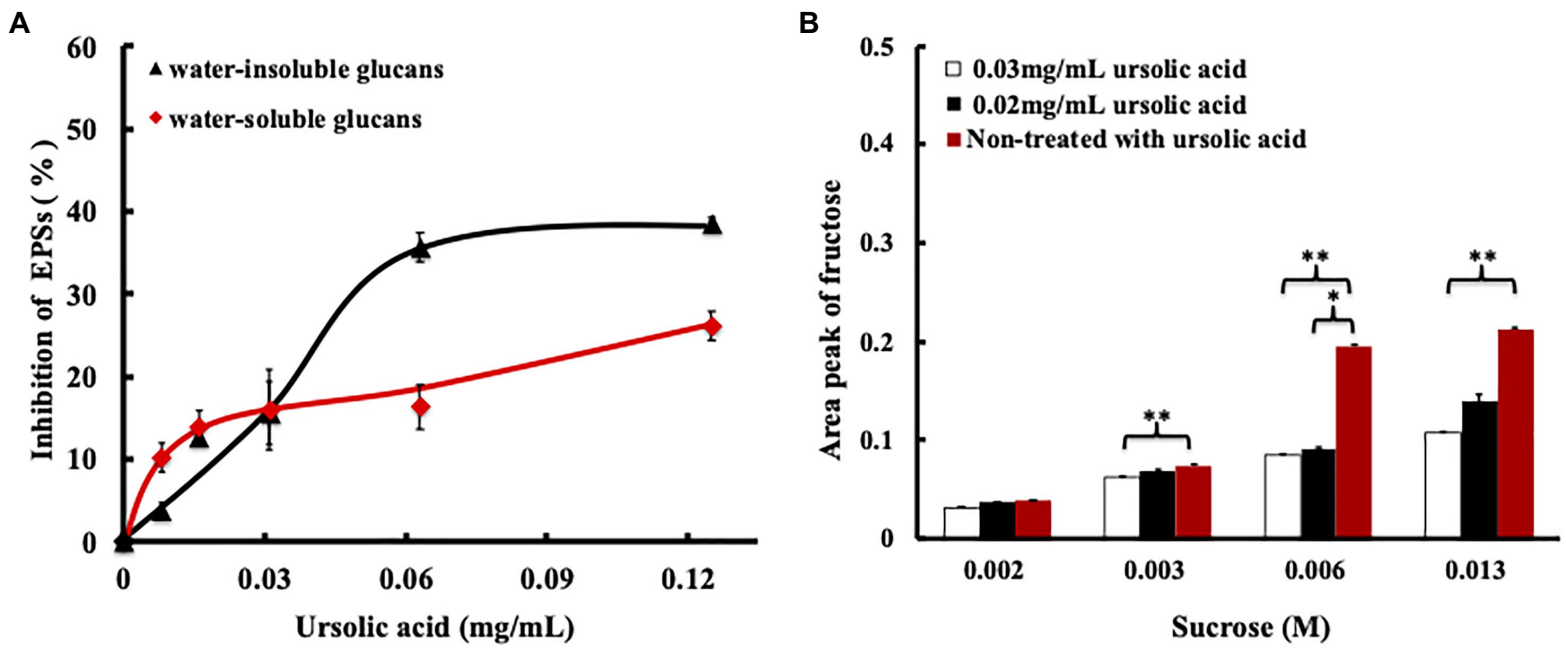
Effect of ursolic acid on GTF catalyzed products. **(A)** Inhibitory percentage of ursolic acid on EPS production determined by phenol/H_2_SO_4_ analysis. After incubation with ursolic acid for 16h, the water-soluble glucans and water-insoluble glucans were prepared from bacterial culture using the method described above. Phenol/H_2_SO_4_ analysis was used to quantify the two types of EPSs. Data are presented as the mean ± standard deviation; **(B)** Effect of ursolic acid on fructose production by GTFs determined *via* CE assay. Data are presented as the mean ± standard deviation. ^*^p<0.05 and ^**^p<0.01.

The process of GTF synthesis of EPS includes two steps: First, the GTF enzyme binds with sucrose, and then, EPS synthesis is catalyzed. After sucrose binds with subsite+1 of the GTF, the glucosyl group of sucrose dissociates from subsite+1 and then binds subsite-1 of the GTF to form an intermediate that catalyzes EPS production, while the fructosyl group of sucrose dissociates from subsite+1 of the GTF to produce the by-product, namely, fructose (Ito et al., 2011). Therefore, the production of fructose and EPSs would decrease when the binding site and catalytic site of the GTF are occupied by a molecule other than sucrose. Since the amino acids in GTF-SI that are essential for ursolic acid binding are also indispensable for sucrose recognition and since the MD simulation results showed that the binding free energy of GTF-SI to ursolic acid was similar to that of sucrose (Supplementary Table S2), we hypothesized that ursolic acid may suppress GTF activity by competitively blocking sucrose binding. To test this hypothesis, we evaluated the effects of ursolic acid on the production of EPS and fructose. To confirm the effect of ursolic acid on EPS synthesis by *S. mutans*, water-soluble glucans and water-insoluble glucans were obtained from bacterial culture treated with or without ursolic acid for 16h, and the phenol/H_2_SO_4_ method was used to detect the content of both types of EPSs. As shown in Figure 4A, ursolic acid at concentrations of 0.008mg/ml to 0.125mg/ml inhibited the production of both water-soluble and water-insoluble glucans. Additionally, the amount of fructose produced by crude recombinant GTF-SI was further measured in the presence of sucrose and ursolic acid for 2h. As shown in Figure 4B, the amount of fructose production decreased with the addition of ursolic acid to the enzymatic reaction system, and the effect of a high concentration was more obvious than that of a low concentration. Based on this in combination with the data above, we proposed that ursolic acid could compete with sucrose to occupy the catalytic center of the GTF, which then inhibits the enzymes that synthesize EPSs and eventually prevents bacterial adhesion to form biofilms.

## Discussion

Dental plaque is an oral bacterial biofilm that plays a key role in oral diseases (Adler et al., 2013). Biofilm formation enhances bacterial tolerance to drugs or the environment; thus, inhibition of biofilm formation and damage to the biofilm are the effective ways to prevent or cure oral diseases (Chenicheri et al., 2017). *S. mutans* is the principal bacterium that forms biofilms by adhesion to teeth and is considered the most important pathogen for dental caries. In the present study, we investigated the effects of ursolic acid on the growth and cariogenicity of biofilms formed by *S. mutans*. The results indicated that ursolic acid could effectively prevent biofilm formation of *S. mutans* through two pathways: (i) Ursolic acid decreased the viability of planktonic and sessile bacteria; and (ii) ursolic acid inhibited GTF-mediated synthesis of EPSs by competing with sucrose to occupy the catalytic center of the enzyme, which decreased bacterial adhesion and biofilm formation. Therefore, ursolic acid has great potential to be developed as an oral protective or therapeutic drug for oral disease.

Dental caries is a secondary bacterial disease that affects the health and quality of life of half of the world’s population (Hwang et al., 2014). Attention is needed to develop effective prevention and treatment methods. The results of this study indicated that ursolic acid inhibits planktonic *S. mutans*. Previously, it was reported that ursolic acid could have an inhibitory effect on other cariogenic pathogens, such as *Streptococcus sobrius* (do Nascimento et al., 2014). Therefore, ursolic acid may have significant antimicrobial activity against pathogenic bacteria involved in dental caries. Traditionally, fluoride, a common component of oral care products, is used to support oral hygiene and health. However, some studies have found that excess fluoride is unsafe for children and adults, as it may induce color nonuniformity of teeth and loss of potency (Wong et al., 2010). Chlorhexidine, a broad-spectrum antibiotic, is widely used to prevent or cure oral diseases (Charugundla et al., 2015). It was reported that *S. mutans* has some drug resistance against chlorhexidine (López-Jornet et al., 2012). Therefore, it is necessary to develop new effective components to decrease the drug resistance of oral pathogens. Interestingly, ursolic acid showed inhibitory activity on biofilms at 1/2 MIC, similar to chlorhexidine (Supplementary Figure S3). Moreover, ursolic acid has low toxicity and is versatile in terms of its biological activity, evidenced by its antiviral, liver protective, and whitening effects (Zou et al., 2019; Liu et al., 2021). Therefore, ursolic acid has great potential to be used as a lead compound in the development of an effective inhibitor for dental caries and as a protective agent for oral health.

Biofilm formation is a dynamic process that includes adhesion, aggregation, and maturation (Arciola et al., 2012). GTFs can synthesize EPSs by using sucrose as a substrate, which provides an adherent ability for bacterial colonization to promote biofilm formation and development (Hellmuth et al., 2008; Chen et al., 2013). Therefore, GTFs are key pathogenic factors in the induction of dental caries. It has been reported that subsite-1 and subsite+1 are the main partial catalytic centers of GTF-SI for sucrose binding and EPS formation (Ito et al., 2011). The primary bound sucrose is attacked by a proton to induce hydrolysis, and then, the glucosyl group binds to subsite-1 of GTF as an intermediate, while fructose is released from subsite+1 of the enzyme (Ito et al., 2011). Amino acid residues such as Arg475, Asp477, Glu515, Asp588, and Tyr916 construct subsite-1 of GTF for catalyzing glucan formation, while the amino acid residues located at subsite+1, such as Tyr430, Leu433, and Trp517, constitute the critical domains for recognition of the moiety (Ito et al., 2011). The molecular docking analysis showed that ursolic acid is sandwiched by Tyr430 and Asp909 to occupy the catalytic center and that the 3-hydroxyl group of ursolic acid points toward the active center. The experimental results confirmed that ursolic acid could bind with GTFs and that the binding ability disappeared as the action site was replaced by other amino acids. Quantitative analysis of EPSs and fructose showed that ursolic acid could decrease the levels of EPSs and fructose. These results confirm that ursolic acid competes with sucrose to occupy the catalytic center of GTFs, which may lead to the failure of GTFs to use sucrose as a substrate to synthesize EPSs (as shown in Figures 2, 4). In addition, the recognition of the glucosyl moiety of primary sucrose by GTFs and the intermediate formation at subsite-1 are well conserved in other *Streptococcus* species related to dental caries and infective endocarditis, such as *Streptococcus gordonii* (Hellmuth et al., 2008). Therefore, ursolic acid could be developed as an antimicrobial agent for dental caries or *Streptococcus*-related diseases.

## Conclusion

We characterized the mechanism of *S. mutans* biofilm formation inhibition by ursolic acid. More importantly, ursolic acid can bind to GTFs instead of sucrose to interfere with microbial adhesion and aggregation through interactions with seven amino acids (Tyr430, Asp909, Leu433, Leu434, Phe907, Trp517, and Tyr916) of the enzyme, which is necessary for biofilm formation and even destroys mature biofilms, to prevent and cure dental caries and other oral diseases. Thus, ursolic acid has the potential to be developed as a drug or oral cleaning product to protect against and cure oral disease and other GTF-related diseases in the clinic. Finally, Leu433, Leu434, and Phe907 may serve as new target sites to screen inhibitors of GTFs to develop new antimicrobials.

## Data Availability Statement

The data sets presented in this study can be found in online repositories. The names of the repository/repositories and accession number(s) can be found in the article/Supplementary Material.

## Author Contributions

LY, BM, FC, and CZ performed the experiments. LY and SL designed the study, carried out the analysis and interpretation, and drafted and revised the manuscript. HY, YL, and ZL designed the study and analyzed the data. YC, SZ, YX, YJ, WS, LL, and WG assisted with the data analysis. All authors contributed to the article and approved the submitted version.

## Funding

This study was supported by grants from the following foundations: the National Natural Science Foundation of China (no. 81700709), the Fundamental Research Funds for the Central Universities (nos. 135130006, 130029804, 111498001, and 131004006), the Research Foundation of Jilin Provincial Science and Technology Development (nos. 20200602023ZP, 20200901002SF, and 20200404124YY), the Foundation of Jilin Province Development and Reform Commission (no. 2020C015), the funds of the Education Department of Jilin Province (no. JJKH20201177KJ), and the Foundation of Human Resources and Social Security Department of Jilin Province (no. 2020009).

## Conflict of Interest

The authors declare that the research was conducted in the absence of any commercial or financial relationships that could be construed as a potential conflict of interest.

## Publisher’s Note

All claims expressed in this article are solely those of the authors and do not necessarily represent those of their affiliated organizations, or those of the publisher, the editors and the reviewers. Any product that may be evaluated in this article, or claim that may be made by its manufacturer, is not guaranteed or endorsed by the publisher.

## Supplementary Material

The Supplementary Material for this article can be found online at:

https://www.frontiersin.org/articles/10.3389/fmicb.2021.743305/full#supplementary-material
