# Supplementary material for: Ursolic Acid Targets Glucosyltransferase and Inhibits Its Activity to Prevent Streptococcus mutans Biofilm Formation
Source: Front Microbiol. 2021 Sep 27;12:743305. doi: 10.3389/fmicb.2021.743305 (PMC8503646; doi:10.3389/fmicb.2021.743305)
Supplement: Supplementary file 1 [file Data_Sheet_1.PDF]

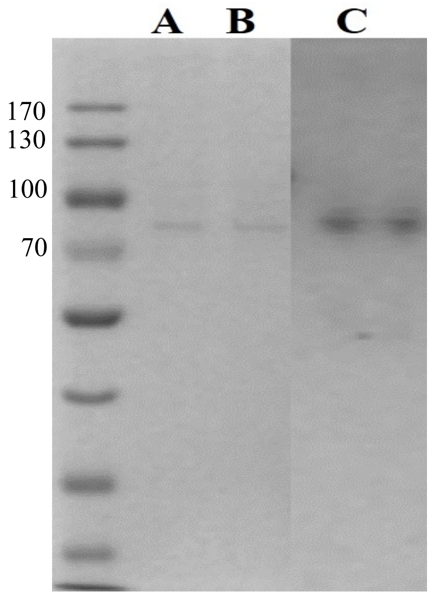

Fig. S1 The GTF-SI and its variant were analyzed by SDS-PAGE.

A: GTF-SI; B:Variant A; C:Varaiant B

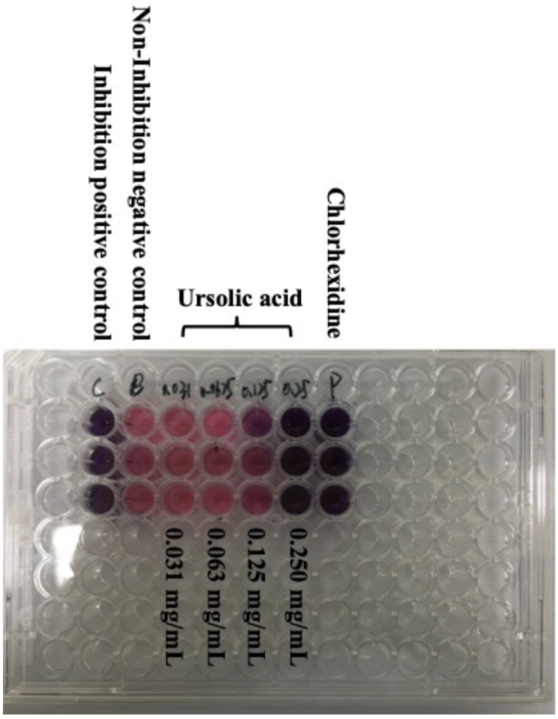

Fig. S2 Determination of MIC for ursolic acid against *S.mutans*. The MIC value is the concentration that could prevent the color from changing to pink.

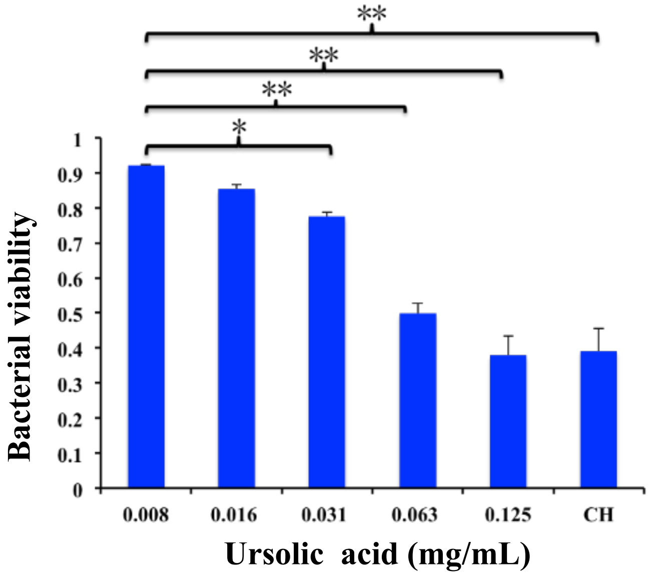

Fig. S3 Effects of ursolic acid on biofilm by XTT assay.

CH: chlorhexidine (the positive control).
