# Supplementary material for: Ursolic Acid Targets Glucosyltransferase and Inhibits Its Activity to Prevent Streptococcus mutans Biofilm Formation
Source: Front Microbiol. 2021 Sep 27;12:743305. doi: 10.3389/fmicb.2021.743305 (PMC8503646; doi:10.3389/fmicb.2021.743305)
Supplement: Supplementary file 2 [file Table_1.DOC]

Table S1 Mutant design of amino acid through site-directed mutagenesis

| Protein | Mutant site | | | | | | |
| --- | --- | --- | --- | --- | --- | --- | --- |
| GTF-SI | Tyr430 | Leu433 | Leu434 | Trp 517 | Phe907 | Asp909 | Tyr916 |
| Variant A | Ala430 | Ala 433 | Ala 434 | Ala 517 | Tyr 907 | Asn 909 | Ala 916 |
| Variant B | Tyr430 | Leu 433 | Leu 434 | Trp517 | Tyr 907 | Asn 909 | Ala 916 |

Table S2 Binding free energy between GTF-ST and mutants and the ligand as determined by MD stimulations

| **Protein** | **ligand** | **Absolute value of Binding free energy** |
| --- | --- | --- |
| GTF-SI | Sucrose | 20.7198 |
|  | Ursolic acid | 21.1594 |
| Variant A | Ursolic acid | 7.6837 |
| Variant B | Ursolic acid | 12.873 |

Table S3 The scoring function of molecular docking between ursolic acid and GTF-SI and its variants

| Protein | ASP score | Gold score |
| --- | --- | --- |
| GTF-SI | 28.5 | 52 |
| Variant-A | 8 | 37 |
| Variant-B | 22 | 39 |
